# Metal-free sampling methods for dust, rainwater, surface water, plants, and sediments: A selection of unique tools from the SWAMP laboratory

**DOI:** 10.1016/j.mex.2023.102521

**Published:** 2023-12-20

**Authors:** Tommy Noernberg, Taylor Bujaczek, Chad W. Cuss, William Shotyk

**Affiliations:** a Department of Renewable Resources, University of Alberta, 348B South Academic Building, T6G 2H1, Edmonton, AB T6G 2R3, Canada; b Current affiliation: School of Science and the Environment, Grenfell Campus, Memorial University of Newfoundland, Corner Brook, NL A2H 5G4, Canada; c Bocock Chair for Agriculture and the Environment, Department of Renewable Resources, University of Alberta, Edmonton, AB T6G 2H1, Canada

**Keywords:** 3D-printer design and fabrication of metal-free devices and tools to minimize contamination by trace elements during sampling of environmental media, Trace elements, Environmental quality, Devices and tools, Contamination control, Metal-free materials, Ultraclean sampling techniques

## Abstract

Contamination control remains one of the greatest challenges for the reliable determination of many trace elements in environmental samples. Here we describe a series of metal-free sampling devices and tools designed and constructed specifically to minimize the risk of contamination by trace elements during sampling of dust, rainwater, surface water, plants, and sediments. Plastic components fabricated using 3-D printing include polylactic acid (PLA), polyethylene terephthalate (PET), polyethylene terephthalate glycol (PETG), polypropylene (PP), polycarbonate (PC) and PC with carbon fibre. When additional strength is needed (e.g. supporting structural components), carbon fibre, aluminum (Al), or 316 stainless steel (SS) is used. Other plastics employed include acrylic and vinyl. Epoxy glue or SS may be used for joining components, but do not come into contact with the samples. Ceramic (zirconium dioxide) cutting blades are used where needed. Each plastic material was evaluated for contaminant trace elements by leaching with high purity nitric acid in the metal-free, ultraclean SWAMP laboratory. The devices were tested in the field to evaluate their performance and durability. When combined with appropriate cleaning procedures, the equipment enables ultraclean collection for trace element analysis of environmental media.

• Plastic sampling devices were designed and constructed using 3D printing of PLA, PET, PETG or PP.
• Leaching characteristics of plastic components were evaluated using high purity nitric acid in a metal-free, ultraclean laboratory.
• Each sampling device was successfully field-tested in industrial settings (near open pit bitumen mines and upgraders), and in remote locations of northern Alberta, Canada.

## Graphical abstract

**Figure:**
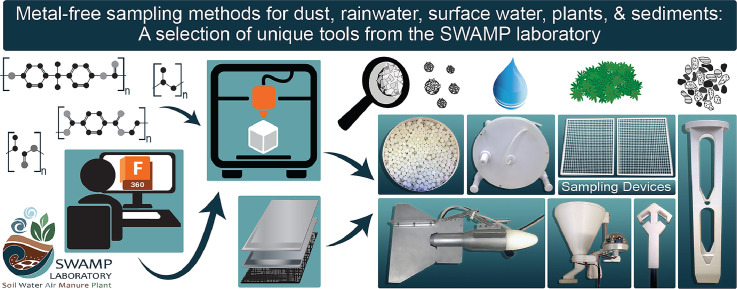

Specifications table

**Table:** 

Subject area:	Chemistry
More specific subject area:	Trace Elements
Name of your method:	3D-printer design and fabrication of metal-free devices and tools to minimize contamination by trace elements during sampling of environmental media
Name and reference of original method:	Not applicable.
Resource availability:	*Metal Alloys* Aluminium 6061 Pole/Rod https://www.mcmaster.com/products/aluminum/material∼6061-aluminum/multipurpose-6061-aluminum-rods-and-discs-7/?s=aluminum+rod+6061 Anodized 6560 Aluminium Framing https://www.mcmaster.com/products/poles/press-fit-framing-and-fittings/?s=aluminum+pole Perforated Strips https://www.mcmaster.com/products/perforated-strips/ 316 Stainless Steel Socket Head Screws https://www.mcmaster.com/products/screws/super-corrosion-resistant-316-stainless-steel-socket-head-screws/ 316 Stainless Steel Philips Flat Head Screws https://www.mcmaster.com/products/screws/head-type∼flat/316-stainless-steel-slotted-flat-head-screws/ 316 Stainless Steel Button Head Hex Drive Screws https://www.mcmaster.com/products/screws/head-type∼rounded/316-stainless-steel-button-head-hex-drive-screws/?s=316+stainless+steel+screws Stainless Steel Twist-Resistant Rivet Nuts https://www.mcmaster.com/products/rivet-nuts/?s=stainless+steel+rivet+nuts *Plastics* Polyethylene Terephthalate Glycol 3D Printer Filament https://www.amazon.ca/SUNLU-Filament-Toughness-Filaments-Printing/dp/B0B99PCLYN/ref=sr_1_8?crid=3QWHO6SWIAMU1&keywords=petg%2Bfilament%2B1.75&qid=1701197750&sprefix=petg%2B%2Caps%2C116&sr=8-8&th=1 Polypropylene Balls https://www.mcmaster.com/products/polypropylene/polypropylene-balls/ Clean Room Fittings https://www.smcusa.com/products/kp-clean-one-touch-fitting-for-blowing-systems∼167019?partNumber=KPH04-02 Polypropylene Nose Cone https://www.mcmaster.com/products/polypropylene/polypropylene-rods/ *Hardware* Acrylic tubing https://www.mcmaster.com/products/tubing/clear-scratch-and-uv-resistant-acrylic-tubes/?s=acrylic+tubing Adhesive Velcro https://www.mcmaster.com/products/hook-and-loop/super-adhesive-back-hook-and-loop-4/ Anycubic Predator https://www.anycubic.com/products/anycubic-predator-fdm-printer Carbon fiber tubing https://www.amazon.ca/Wrapped-Carbon-Glossy-Surface-810170mm/dp/B08KZMW7JT/ref=sr_1_6?crid=5340TSAFOJG4&keywords=carbon%2Bfiber%2Btubing&qid=1694525749&sprefix=carbon%2Bfiber%2Btubing%2Caps%2C128&sr=8-6&th=1 Ceramic cutting blades https://www.mcmaster.com/products/blades/utility-knife-blades-5/?s=ceramic+blades Dakota Lithium 12B 10Ah LiFePO4 Battery https://www.cabelas.ca/product/130371/dakota-lithium-12-volt-10-ah-battery Hydreon Optical Solid State Rain Gauge Model RG 9 https://rainsensors.com/products/rg-9/ Solar Charge Controller LS1012EU 10A 12V https://www.amazon.ca/Controller-LS1012EU-Regulator-Indicator-Charging/dp/B079HRH1B6 Solar panel https://www.amazon.ca/ECO-WORTHY-20W-Solar-Panel-Polycrystalline/dp/B00PFGP0EA/ref=sr_1_2?crid=2GNH5VHMYAR69&keywords=solar%2Bpanel&qid=1689695324&rnid=116441864011&s=lawn-garden&sprefix=solar%2Bpanel%2B%2Caps%2C117&sr=1-2&ufe=app_do%3Aamzn1.fos.b06bdbbe-20fd-4ebc-88cf-fa04f1ca0da8&th=1 Technical Air Products HEPA Fan Filter Unit https://www.technicalairproducts.com/cleanrooms-components/fan-filter-units/ Vinyl https://www.mcmaster.com/products/vinyl/clear-chemical-resistant-pvc-film-6/ 12 V 10Amp Forward and Reverse Relay Module https://czh-labs.com/products/12v-10amp-forward-and-reverse-relay-module-for-motor-linear-actuator-reversing-relay-module?gclid=CjwKCAjwhdWkBhBZEiwA1ibLmAr0lTAX5Yc5EiWP6NDdBX-VLaFMGkWwLZER8a_Ov14zPjLEQfMmNxoCRHEQAvD_BwE 316 Stainless Steel Plates for FISH. https://www.mcmaster.com/products/∼/material∼316-stainless-steel/corrosion-resistant-316-stainless-steel-6/?s=316+stainless+steel
	*Software* Cura slicing software https://ultimaker.com/software/ultimaker-cura/ Fusion 360 https://www.autodesk.ca/en/products/fusion-360/overview?mktvar002=5022376|SEM|15561023979|131875405835|kwd-11029869505&utm_source=GGL&utm_medium=SEM&utm_campaign=GGL_D-M_Fusion-360_AMER_CA_eComm_SEM_BR_NEW_EX_0000_5022376_F360&utm_id=5022376&utm_term=kwd-11029869505&mkwid=s|pcrid|647628303821|pkw|fusion%20360|pmt|e|pdv|c|slid||pgrid|131875405835|ptaid|kwd-11029869505|pid|&utm_medium=cpc&utm_source=google&utm_campaign&utm_term=fusion%20360&utm_content=s|pcrid|647628303821|pkw|fusion%20360|pmt|e|pdv|c|slid||pgrid|131875405835|ptaid|kwd-11029869505|&gclid=CjwKCAjwv8qkBhAnEiwAkY-ahgmb-hgOf_8CPH5mtL-LzXU2qX1jfztTfGitkRmeelWtCpV70dL7zxoCgBEQAvD_BwE&ef_id=ZJN7pQAABUajuEO1:20230621223725:s&term=1-YEAR&tab=subscription&plc=F360

**Method details**

## Introduction and background

The study of trace elements (TEs) in the environment presents a number of technical challenges for the geochemist, for two main reasons. First, the concentrations of TEs in the samples of interest may be very low, requiring great analytical sensitivity for their reliable determination. Abundances of TE in soils are commonly in the mg/kg range, but in plants they may be hundreds or thousands of times lower. For example, in *Sphagnum* moss collected in remote areas of northern Alberta, Canada, Ag, Be, Cd, Sb, and Tl are in the µg/kg range [1]. In natural waters, the challenges may be greater still, with these and other TEs including Cr and Pb present in the range of ng/l or below [2]. However, the problem of analytical sensitivity has largely, although not entirely, been overcome with the development, commercial introduction and widespread use of inductively-coupled plasma – mass spectrometry [3,4]. The second major problem is the risk of contamination during sample collection as well as handling and processing in the laboratory. Early efforts to identify, quantify, and circumvent sample contamination have been summarized in pioneering works [5], [6], [7], [8], [9], [10]. In this regard, work in the laboratory of the late Clair Patterson at CalTech, deserves special consideration. For example, consider his pioneering study of three thousand years of atmospheric Pb deposition in Greenland ice which introduced the concept of global environmental contamination by this toxic metal [11]. Today we recognize that contamination control and the establishment of low, reproducible blank values, represents the limiting factor in TE analysis of environmental samples [12], [13], [14], [15]. Water samples have presented some of the greatest challenges and have probably received the most attention [16], [17], [18], [19], [20], [21], [22], [23], [24]. Many of the most significant problems regarding sample handling and processing have been overcome through the design and construction of metal-free, ultraclean laboratories [25], [26], [27].

Biological samples present an additional challenge in that they may be contaminated by the medium in which they live [6,8]. For example plant matter is commonly contaminated by soil-derived mineral particles [28], [29], [30]. Several procedures for washing plants have been developed to remove dust particles from leaves in order to measure TE uptake from soil, for example [28]. However, due to the differences in plant structures, leaf surface morphology and waxy cuticle composition, many dust particles remain trapped on the plant surface even after washing [31]. Similarly, water samples may contain abundant particulate matter in the form of suspended solids from erosion and transport, especially in surface waters [32] which can be especially pronounced in high elevation regions, following precipitation events, and during spring snowmelt [33,34]. The problem presented by mineral particles, however, whether they occur in plant or water samples, can be circumvented by considering element ratios whereby the TEs of interest (or environmental concern such as As, Cd, Pb, Sb or Tl) are normalized to the concentrations of TEs that serve as chemical surrogates for the abundance of insoluble mineral matter. Specifically, these refractory elements include Al, Hf, Th, Ti, and the rare earth elements i.e. Sc, Y, and the lanthanides [35]. These elements are neither essential to plants [36] nor animals [37,38] and, therefore they are not subjected to active biological uptake. However, the sample collection device itself, commonly represents the first risk of TE contamination. Many of the materials used to construct sampling devices, even many plastics, and present contamination risks due to abrasion or leaching (SI Table S1). Construction materials used to build corers for collecting lake sediments commonly include aluminum alloys, other non-ferrous metals, and steel (SI Table S2) [39,40] which introduce risks of TE contamination, although plastic is increasingly employed [41,42]. These concerns illustrate the importance of material selection and the design of sampling devices and tools, for limiting the risk of contamination by TEs during sampling.

Here, we introduce and describe some of the metal-free devices we have developed in the SWAMP laboratory, a metal-free, ultraclean laboratory for the study of TEs in Soil, Water, Air, Manure, and Plants. These tools were designed to reduce the risk of TE contamination during sampling of dust, rainwater, surface waters, plants and sediments (SI Table S2). The devices are novel designs that are not commercially available, and are intended reduce the risk of TE contamination by orders of magnitude, compared to metal tools. Ideas and technical information are freely shared here, so that others may profit from this experience and perhaps even make improvements. Additional approaches that we have developed over the years to minimize contamination by TEs while sampling *Sphagnum* moss [43], peat [44], snow [45], surface waters [46], and groundwaters [2] are described in those publications.

### Design and fabrication

The Fusion 360 drawing program was used to conceptualize each plastic component. The components were then fabricated using an Anycubic Predator and QIDI Tech (X-max) 3-D printer with Cura slicing application software. Polylactic acid (PLA) was often used to create prototypes design evaluation or for demonstration purposes. Polyethylene terephthalate (PET), polyethylene terephthalate glycol (PETG), polypropylene (PP), polycarbonate (PC) and PC with carbon fibre, were used to construct the main components. To minimize the number of drilled holes for threading screws, the holes were 3-D printed whenever possible. Metal components needed for structural rigidity or support are made either of aluminum alloys or 316 stainless steel. Aluminum alloys are lightweight, strong, and resistant to corrosion. Moreover, Al is typically the most abundant TE in soil, plant and water samples, thus limiting the risk of relevant blank contributions. The 316 stainless steel alloy was shown long ago to be suitable for the chisels needed to decontaminate ancient samples of Greenland ice while avoiding Pb contamination [11], and more recently for sampling pristine groundwater containing even lower Pb concentrations [2].

### Aeolian frisbee dust collector

**Fig. 1:**
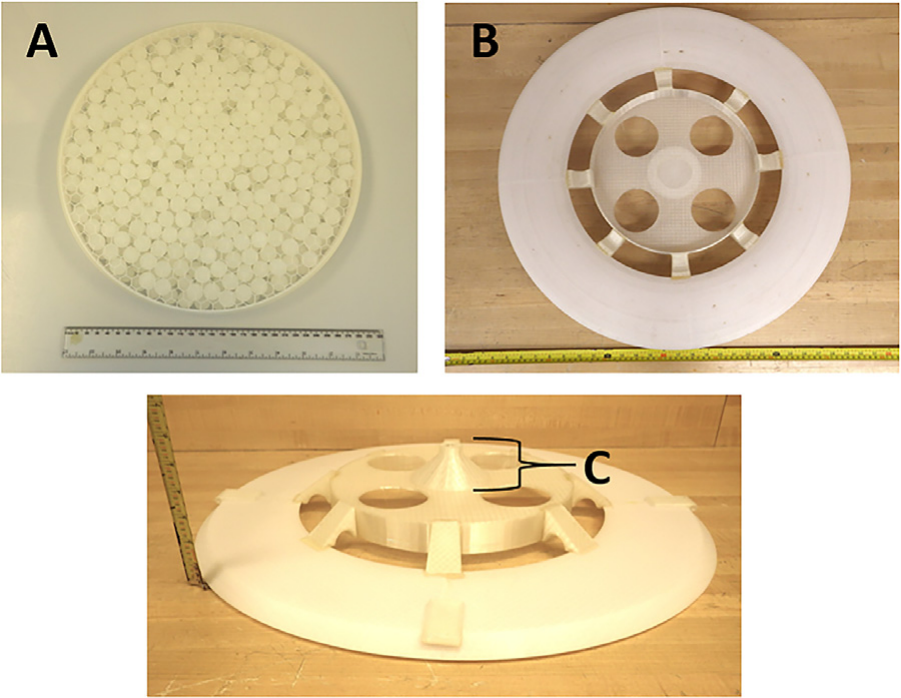
Aeolian Frisbee dust collector collecting bowl (a) and dorsal (b) and ventral (c) perspectives of the deflector rings.

An Aeolian inverted Frisbee dust collector which had previously been used to study sampling efficiency as a function of particle size [47], was constructed using plastic. PETG was used throughout, except where noted. The sampler features a central mount for the collecting bowl surrounded by an aerodynamically shaped deflector ring (diameter = 62.3 cm; height = 9.8 cm) (Fig. 1b). A separate, removable collecting bowl (diameter = 29.7 cm; height = 3.0 cm; wall thickness = 0.1 cm), is filled with two layers of 1.6 cm diameter PP balls (Fig. 1a). Dust accumulates on the PP balls housed in the deflector ring while minimizing the risk of contamination by TEs. A circular grid (diameter = 29.7 cm; height = 2.0 cm; wall thickness = 0.2 cm) is placed over the balls to prevent them from overflowing in the collecting bowl during rainfall. When the removable collecting dish is not deployed or there is rainfall, a lid (diameter = 30 cm; height = 1.0; cm; wall thickness = 0.2 cm) covers the balls (Fig. 1a). The removable collecting bowl is placed inside the central mount attached to the deflector ring, and serves to collect the dust particles (Fig. 1).

The deflector ring also features an outer ring (outer diameter = 64.5 cm; inner diameter 38.5 cm; Fig. 1b). The aerodynamic outer ring gradually tapers towards the central mount which enables the deflector ring to direct air and dust towards the collecting bowl (Fig. 1b). The central mount (diameter = 29.9 cm; height = 3.0 cm; wall thickness = 0.25 cm) is stabilized to the outer ring with eight PETG spokes (3.3 × 6.2 × 3.0 cm) using epoxy glue (Fig. 1b). The central mount features four holes (radius = 8.0 cm) along its base (Fig. 1b). This allows the researcher to deploy and push out and the removable collecting bowl in/out of the deflector ring when it is deployed. Underneath the deflector ring is a mount (diameter = 9.5 cm; height = 4.5 cm) that tapers down to form a hole (outer diameter = 3.0 cm; inner diameter = 1.5 cm; Fig. 1c). An aluminum pole is inserted into this inner hole, allowing the dust collector to be mounted 2 m above the ground surface. The underside of the deflector ring also features four rectangular plastic pieces (2.9 × 5.5 × 0.5) in four compass directions attached with epoxy glue (Fig. 1c). Each of the four plastic pieces has a 0.5 cm hole on one side of the plastic (Fig. 1c), allowing ropes to pass through to secure the dust collector to the ground (Fig. 1c).

### Moss boxes for dust collection

**Fig. 2:**
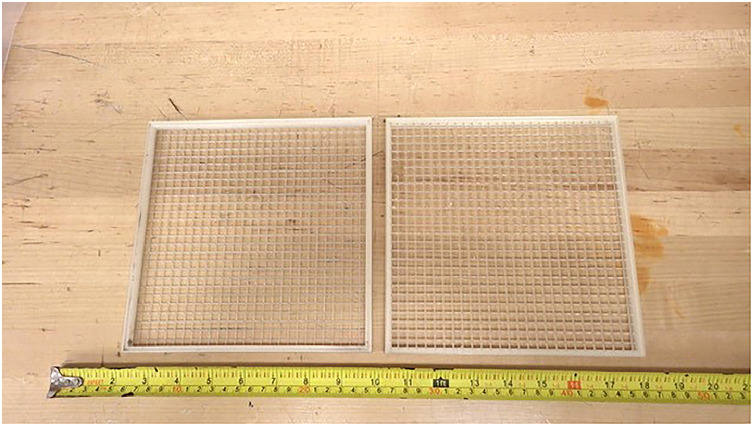
Metal-free boxes showing the two identical halves that store *Sphagnum* moss for collecting dust particles and aerosols for TE analysis.

Moss has long been used as a biomonitor of atmospheric deposition of TEs because of its unique physical properties, in particular its large surface area and cation exchange capacity [48]. Containers filled with moss, known as moss bags, have been used to study pollution from diverse sources [49], [50], [51]. Here, metal-free boxes were created using two male and female PETG grids (20 × 20 × 1 cm with 0.6 cm mesh) to permit air and dust flow across *Sphagnum* moss packed into these containers (Fig. 2). The two halves of the boxes are secured further using plastic cable ties at the four corners (Fig. 2).

### Moisture activated rain collector

**Fig. 3:**
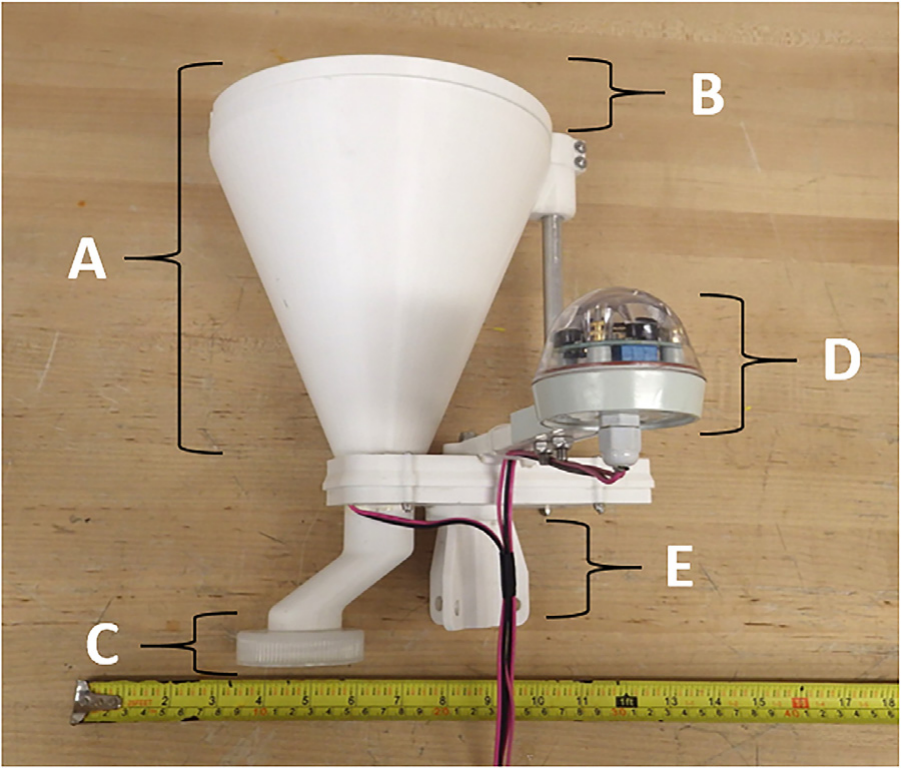
Moisture activated rain collector: (a) funnel, (b) funnel lid, (c) moisture activation sensor, (d) cap for collection bottle, and (e) attachment for mounting on a pole.

Avoiding contamination during rainwater collection is crucial, given the very low “background” concentrations of TEs in precipitation, and the need for accurate information about deposition rates [52,53]. A metal-free, moisture activated rain collector was designed and constructed in PETG (except where noted) to minimize contamination by TEs during sample collection (Fig. 3). The rain collector consists of six parts: (a) a funnel, (b) a lid that spans the large opening of the funnel, (c) a 500 mL PP lid for the collection bottle, (d) a moisture activated sensor (e) a plastic cylinder to attach a pole, (Fig. 3) and (f) a power supply consisting of a solar panel, a PWM Solar Charge Controller (switched to lithium mode; Model No. LS1012EU 10A 12 V), a 12 V 10 Amp Forward and Reverse Relay Module (Model F-1020), and a Dakota Lithium 12B 10Ah LiFePO4 battery.

The rainwater collector (29.2 × 31 × 32 cm) is deployed on a 2 m high aluminum pole (Fig. 3). Plastic components are secured with 316 stainless steel screws (Fig. 3). The attachment for the 1.3 cm diameter pole includes a circular platform (5.4 × 5.4 × 0.8 cm) that secure it to the main body of the rain collector (Fig. 3e). A hollow plastic cylinder (6.0 × 2.5 × 2.5 cm) is attached to the platform and features three wings (6.0 × 2.0 × 0.6 cm; Fig. 3e). Each wing has a 1 cm hole allowing ropes to pass through and secure the rainwater collector to the ground (Fig. 3e). The funnel (18.5 × 22.8 × 22.8 cm) is fixed using epoxy glue to a main platform (17.0 × 8.4 × 2.4 cm) and features a hollow cylinder near the wide opening (3.0 × 3.0 × 3.0 cm; Fig. 3a). The epoxy glue is not exposed to the water sample passing through the funnel (Fig. 3a). The cylinder near the wide opening of the funnel allows a small aluminum pole (20.6 × 1.3 × 1.3 cm) to be inserted, secured to the main horizontal platform, and reinforces the funnel's position (Fig. 3a).

The funnel lid (diameter = 22.8 cm; height = 1.0 cm) is placed horizontally on the funnel and the moisture activated sensor (Hydreon Optical Solid State Rain Gauge Model RG-9) controls the rotation of the pole attached to the lid, allowing the lid to open and close (Fig. 3). The moisture activated sensor is mounted on a 16.0 × 2.0 × 1.0 cm stand attached to the main platform (Fig. 3d). In the absence of rainfall, the lid on the funnel remains closed (Fig. 3b; c). When raindrops start to fall and land on the sensor, the infrared light produced inside the sensor is allowed to pass through the plastic lens [54] The sensor detects the change in light intensity and a switch is turned on to open the funnel lid (Fig. 3d). The lid opens by swinging horizontally by 180°. A timer keeps the funnel lid open for 15 min after rainfall has ceased, and closes automatically. Moisture activation allows the rainwater collector to be opened and closed automatically, eliminating the need to be present at the sampling site while limiting contamination from dust particles to the collection bottle. The moisture activated sensor (Fig. 3) is powered by a lithium battery housed in a waterproof case (Pelican Protector Case^TM^, Torrance, CA, USA). Electrical wires connect the moisture activation switch to the relay, which is connected to the PWM Solar Charge Controller (Fig. 3f). A 42 × 32 cm solar panel on two aluminum poles is positioned at a 45° angle facing the sun's path of travel. Solar energy charges the lithium battery controlled by the PWM Solar Charge Controller, and transfers power to the relay, allowing the rainwater collector funnel lid to open and close (Fig. 3f).

Rainwater falls into the funnel and is directed into the acid-cleaned, PP collection bottle (Fig. 3c). Rainwater does not come into contact with the epoxy glue or the 316 stainless steel screws during collection. A 3.6 cm wide hole is created at the narrow end of the funnel, through the main platform, and passes a channel down to the collection bottle (Fig. 3c). The channel from the funnel to the collection bottle extends 45° away from the main platform allowing space for a large 1 L PP bottle to be attached. The collection bottle lid has a hole cut through, and rests on a 5.6 × 5.6 × 0.3 cm ledge allowing bottles to be changed out with ease (Fig. 3c).

### Water column sampler

**Fig. 4:**
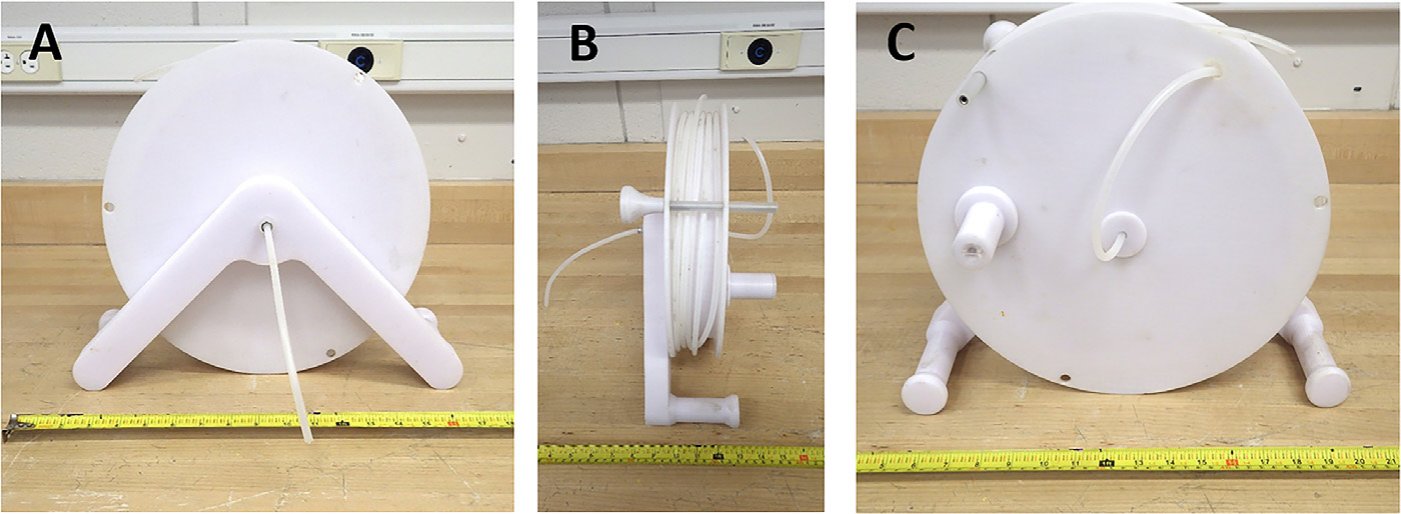
(a) Water column sampler demonstrating the PE tubing that transports water into a sampling bottle, (b) a profile of the sampler, and (c) the reverse side of the wheel, demonstrating the handle that turns the wheel and lowers the tubing to the desired depth.

Conventional water sampling for TEs emphasizes spatial and temporal variation in surface samples [42], but provides no information about changes with regard to depth. To fill this gap, a metal-free water column sampler was fabricated using PETG (Fig. 4). This device features two identical 32.5 × 32.5 × 0.4 cm wheels with three 1 cm holes placed equidistant (Fig. 4). The two wheels are attached to a wheel smaller in diameter (27.5 cm; height = 0.4 cm) on the outside of each face (Fig. 4b). This creates a 5 cm wide channel to wrap acid-cleaned polyethylene (PE) tubing around the wheel (Fig. 4b). The wheel is mounted on a stand (29 × 3.0 × 4.0 cm) that rests on two plastic cylinders (11.7 × 4.0 × 4.0 cm) that allows the wheel to be stabilized perpendicular to its surface (Fig. 4a; b).

The water column sampler collects water samples into a bottle using a vacuum. A 1 cm hole is drilled in the back of the wheel along the border (Fig. 4c). PE tubing from the wheel is fed through this hole to a second hole located centrally on both sides of the wheel (Fig. 4a; c). The PE tubing is fed out of the front of the wheel where the wheel mount is located and secured with clean room fittings to ensure a clean connection between the hose and sampling device; Fig. 4a). This tubing is attached to a collection bottle lid with a 1 cm hole drilled through. A second hole is drilled though the lid and a shorter piece of PE tubing is pushed through the hole. A vacuum is created in the collection bottle with a peristaltic pump, and draws water into the collection bottle (Fig. 4a). To sample water, the PE tubing wrapped around the wheel is lowered into the water column using a cylindrical handle (5.1 × 2.8 cm) on the back of the wheel (Fig. 4c). The handle is hollow and is inserted onto a rod (5.8 × 2.8 cm) on a circular platform (diameter = 4.0 cm; height = 0.8 cm) on the back of the wheel (Fig. 4c). Pushing the handle down allows the wheel to turn without adjusting hand position, and the PE tubing to be lowered seamlessly (Fig. 4c). The distance between the holes on the wheel border is 30 cm, allowing the researcher to verify sampling depth once the tubing is lowered (Fig. 4a; c). To prevent the tubing from floating, an 316 stainless steel weight is used: this is encased in several layers of 3-D printed PP with a hole running through the center. PE tubing is fed through the weight and secured using a clean room fitting. To maintain water sampling at the desired depth, a small pole with a handle (14 × 3.4 cm at its widest) is pushed through both holes of the wheel (Fig. 4b). This secures the wheel, and prevents the PE tubing from being lowered any further (Fig. 4b). The PE tubing length wrapped around the wheel is 6 m, but can easily be lengthened to the desired sampling depth. The water column sampler is ideal for lentic freshwater habitats and can be used on a watercraft or over ice.

### SWAMP Lab “Fish”

**Fig. 5:**
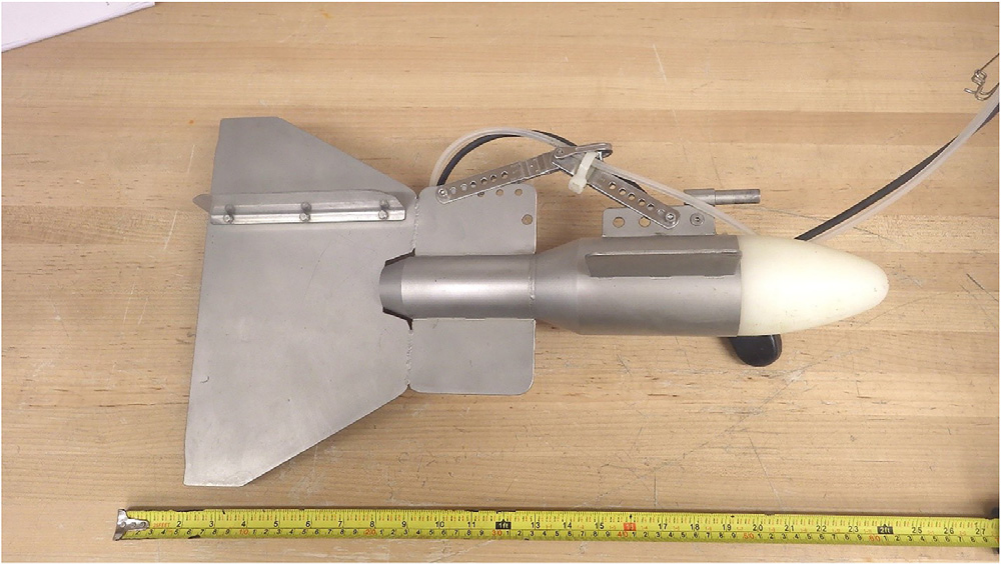
SWAMP Lab “Fish” with a plastic inlet cone that faces upstream during river water sampling.

The SWAMP Lab “Fish” is an ultraclean torpedo water sampler ideal for sampling rivers or streams at various depths (Fig. 5). The “Fish” (55.5 × 19.5 × 32.0 cm) features a solid polypropylene rod (not Teflon as indicated earlier [34]) which is 10.5 × 8.0 × 8.0 cm and turned on a lathe to form a distinctive cone shape. A 0.6 cm wide hole is drilled through the dorsal side and exits through the tip of the nose cone. Acid-cleaned PE tubing passes through this hole and extends out 5 mm past the tip of the nose to ensure that the tubing comes into contact with the water upstream of the “Fish” sampling device. When the “Fish” is deployed, the nose cone faces upstream and a peristaltic pump is used to sample water at the desired depth into an acid-cleaned bottle. The nose cone fits into a 316 stainless steel cylinder (27.5 × 8.0 × 8.0 cm) to give it weight (Fig. 5). The cylinder features two horizontal, 316 stainless steel fins (10.1 × 6.0 × 0.3 cm), and a third, vertical 316 stainless steel fin (8.8 × 0.3 × 2.5) with four 1 cm holes (Fig. 5).

The “Fish” features two additional, larger 316 stainless steel fins that serve to stabilize the nose cone under high currents (Fig. 5). The larger fin (28.1 × 0.3 × 32.0 cm) is attached perpendicular to the river when the nose cone is directed upstream, and the smaller fin (17.7 × 4.4 × 0.3 cm) is attached parallel to the river bottom (Fig. 5). The larger fin features five holes on the dorsal square portion, and two 11.5 cm 316 stainless steel perforated strips are attached with 316 stainless steel screws to the larger square fin, and to the nose cone (Fig. 5). The PE sampling tubing is secured to the perforated strip using a cable tie (Fig. 5). The angle of the perforated strips can be changed by adjusting their placement on the nose cone fin and the large square fin. This allows for the torpedo sampler to stay level as the current and depth changes (Fig. 5). The “Fish” is deployed into the water column from a boat into a river or stream and can collect water from any depth [34].

### Laminar flow, clean air growth chambers

**Fig. 6:**
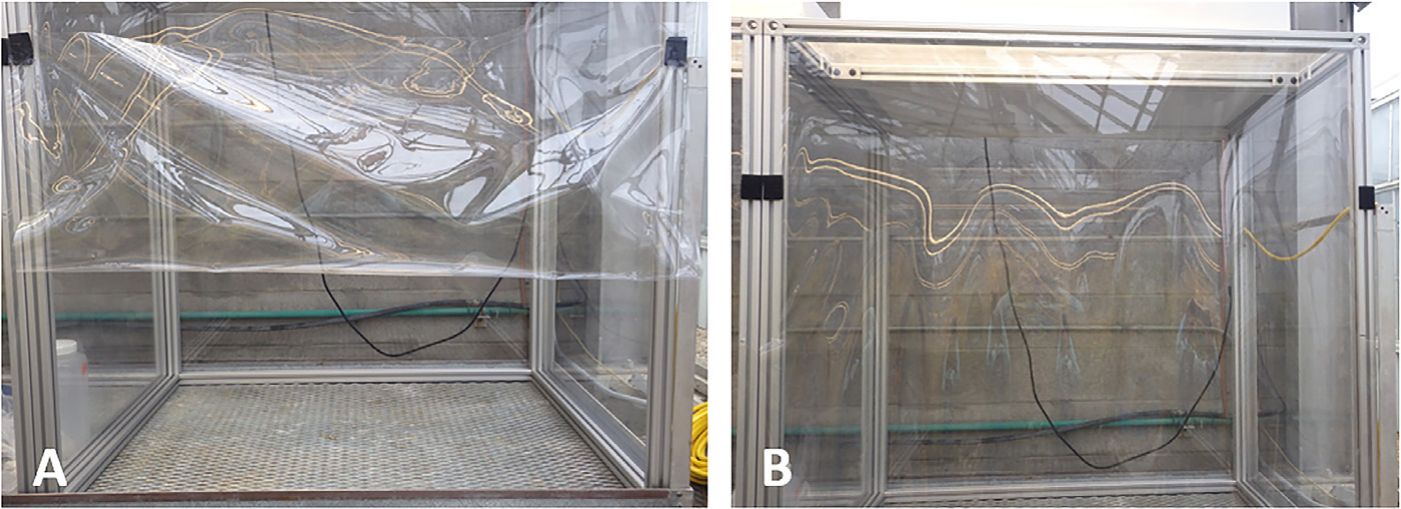
Laminar flow, clean air growth chambers. The transparent plastic cover is secured high on the cabinet (a). With the plastic cover lowered, there is laminar flow of HEPA filtered air within the chamber (b) from the inside to the outside, preventing the entry of ambient air.

Plants sampled in their natural habitat have soil-derived mineral particles on their surfaces, and this renders it very difficult to distinguish between uptake of TEs from soil versus aerial deposition [28], [29], [30]. To allow true plant uptake of TEs to be determined, growth chambers were designed and constructed for the University of Alberta Greenhouse using high efficiency particulate air (HEPA) (Fig. 6). The chambers (134 × 131 × 100 cm) are fabricated from three vinyl sheets (132 × 125 × 95 cm) attached to an anodized 6560 aluminum frame (Fig. 6). The opening to the growth chamber is protected by a flexible vinyl plastic cover secured to the top of the cabinet using double-sided tape. The plastic cover is raised and attached to the growth chamber using Velcro to allow cabinet access (Fig. 6a). An air handling unit with adjustable air flow supplies air to the HEPA filter (Technical Air Products) which is installed on top of the growth chamber (Fig. 6). Laminar flow of filtered air moves from the top of the chamber and onto the growing plants resting on a grated table (Fig. 6). A Lighthouse portable hand-held laser particle counter (3016 IAQ) was used to monitor air quality, and for the two and a half month experiments, no dust particles were detected inside the growth chambers (SI Table S3).

### Sampler for submerged aquatic plants

**Fig. 7:**
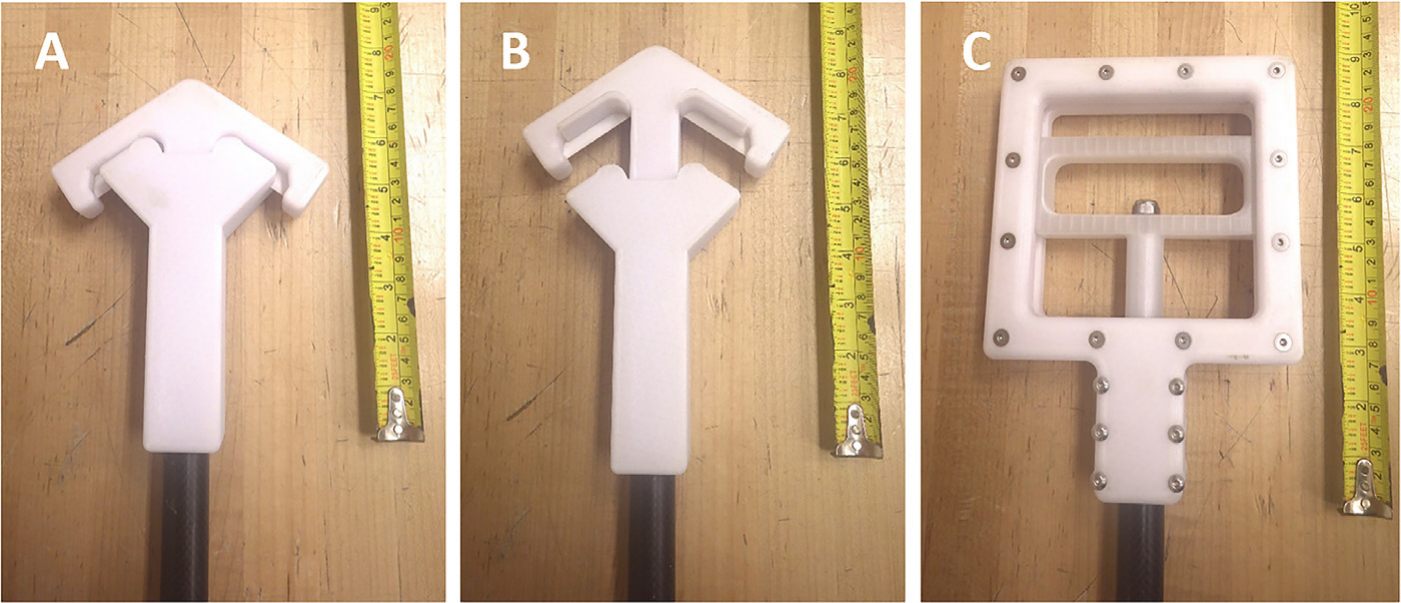
Sampler for submerged aquatic plants. The device in the closed (a) and in the open positions (b) displaying the ceramic blades. The handle is fastened using stainless steel screws which secure it to a carbon fiber pole (c).

A metal-free device was designed and constructed for sampling aquatic plants in shallow surface waters (Fig. 7). The device is 2.34 m long and enables sampling of submerged aquatic plants without entering the water, minimizing disturbance to the sampling location and making it ideal for use on unstable ground such as the shores of ponds and wetlands. The sampler features three components: the handle, the carbon fibre pole, and the cutting end (Fig. 7). The carbon fibre pole has two sections measuring 50.3 cm, connected using epoxy glue to aluminum poles (Fig. 7). The carbon fibre tubing was selected because of its low weight and great strength.

The sampler features a handle (14.9 × 14.9 × 4 cm) printed using PETG in two identical “top” and “bottom” square pieces. The “top” and “bottom” pieces are secured with eighteen stainless steel screws and features a space in the center of the square (11 × 11 cm) (Fig. 7c), allowing for a narrower PETG handle (11 × 1 × 1.8 cm) to fit inside. The narrower handle is secured to an aluminum pole using a stainless steel screw and the pole is inserted inside the carbon fibre tubing (Fig. 7c). The narrow handle is able to shift down 3.5 cm and opens and closes the cutting device which is attached to the opposite end of the interior pole (Fig. 7a; b). The narrow handle can shift down because it rests on a groove (1.2 × 0.5 cm) that runs in between the two plastic “top” and “bottom” square pieces (Fig. 7c).

The cutting end is constructed of a rectangular piece (46 × 3.6 × 4.3 cm) of PETG that encases the carbon fibre pole (2 cm diameter; Fig. 7a; b). The second component of the cutting end is constructed in a diamond shape (7.6 × 2.5 × 2.6 cm) at an angle of 45° and is attached to the pole inserted inside the carbon fibre tubing (Fig. 7a; b). This component is able to shift open and closed by the pole attached to the handle at the opposite end, exposing the ceramic (zirconium dioxide) cutting blades (4.1 × 1.0 cm; Fig. 7). The cutting blades in the closed position fit into two 1 cm slits on the rectangular plastic portion, allowing the blades to remain protected (Fig. 7a). When the sampler is in the opened position, the cutting blades are pushed 3.5 cm away from the pole, allowing a plant sample to fit inside the opening (Fig. 7b). Closing the sampler using the handle allows the plant sample to be cut at the desired height and retrieved without the plant falling out of the sampler. This is accomplished using with two slightly curved plastic pieces (4.5 × 0.4 × 2.4 cm) attached perpendicular to the ceramic cutting blades (Fig. 7b).

### Lake sediment corer

**Fig. 8:**
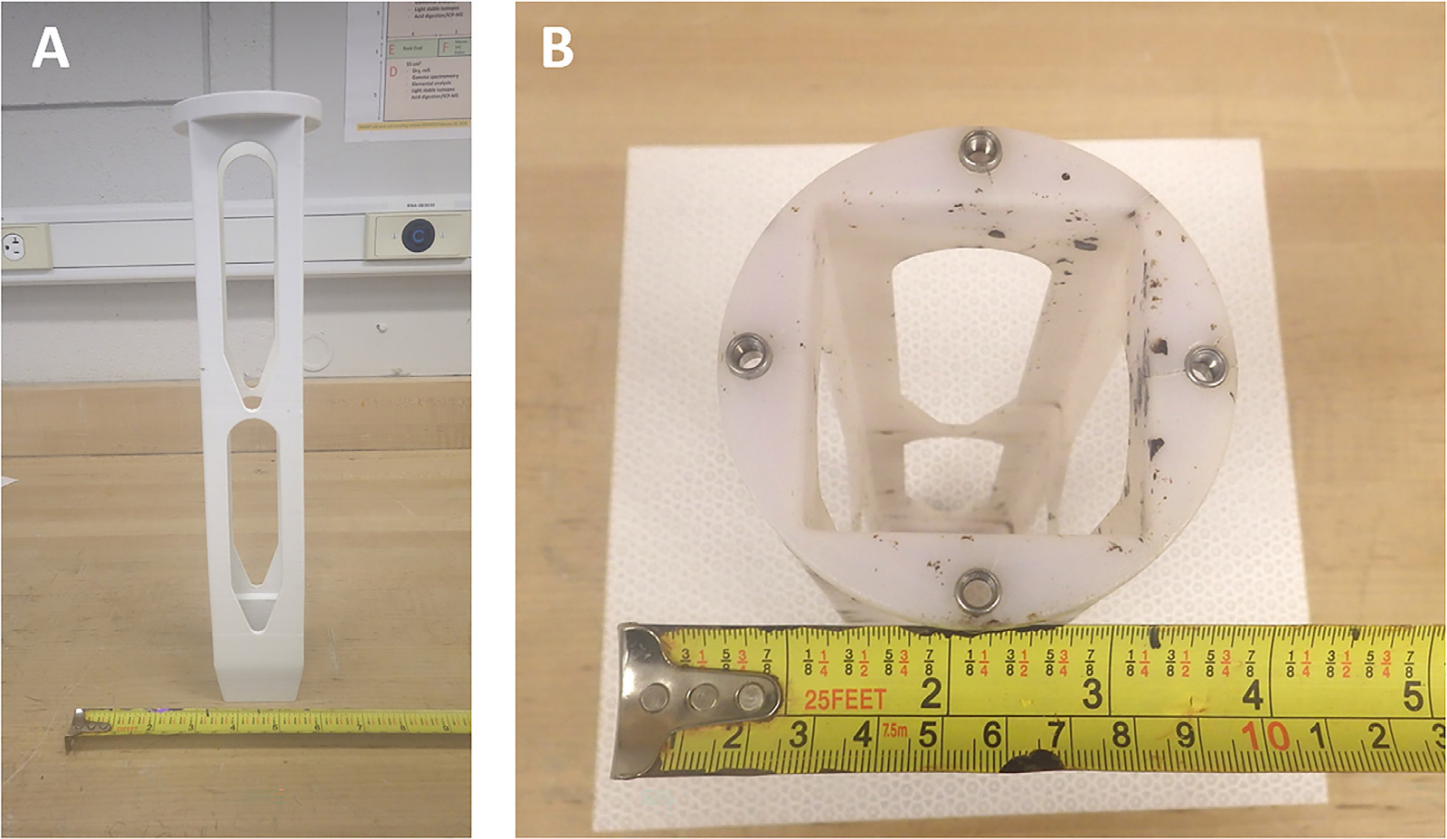
Lake sediment corer featuring (a) the square opening where sediment can enter the sampler and (b) the circular plate where the acrylic tubing is loaded and attached to the sampling pole.

**Fig. 9:**
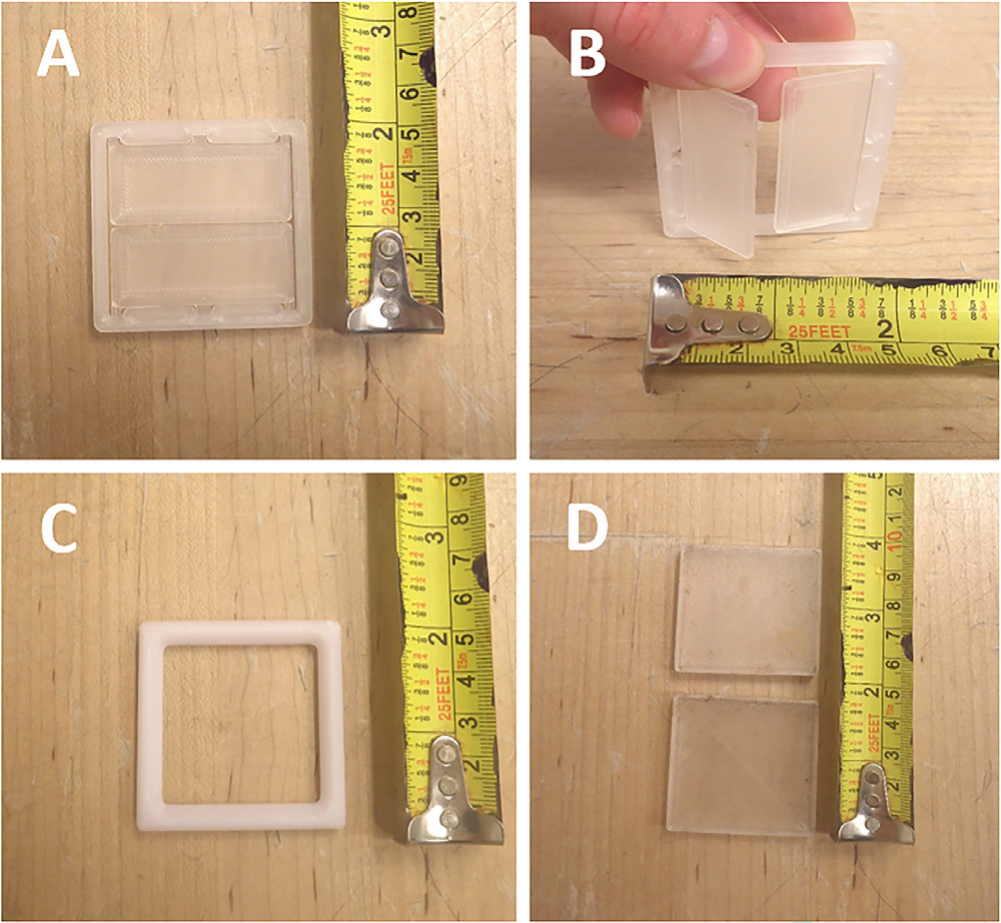
Accessories for the lake sediment corer including (a) the door that rests on the small platform, (b) orientation of the one-way door, (c) hollow square casing that secures the door to the platform, and (d) square plastic that is inserted into both sides of acrylic tubing housing the lake sediment sample.

A metal-free lake sediment corer was developed to collect lake sediments while minimizing contamination from the metal alloys which are used in virtually all of the other sediment coring devices [54] Constructed of PETG, this tool measures 36 × 5.2 × 5.2 cm, and is 0.3 cm thick (Fig. 1). The corer features a tapered square opening measuring 4.2 × 4.2 × 0.3 cm where sediment enters the corer (Fig. 8a). The square opening gradually widens from 4.2 to 5.2 cm along a distance of 3 cm (Fig. 8a). A small platform is created here measuring 0.5 cm in length, and a hollow square casing (5.2 × 5.2 × 0.5 cm) is placed horizontally on the small platform (Fig. 2c). A small PP door (5.2 × 5.2 × 0.5 cm) is inserted horizontally onto the casing, securing the door in place (Fig. 9a). The door features two PP segments (4.3 × 2.0 × 0.5 cm) fabricated on hinges that allows the door to open in only one direction (Fig. 9a; b). Once the corer is pushed down into the sediment, the one-way door serves to keep the corer closed as the corer is raised to the surface, preventing sample loss (Fig. 9b). Acrylic tubing (33.5 × 5.7 × 5.2 cm) is loaded into the corer from the opposite end (Fig. 8a; b). The acrylic tubing rests on the platform, secures the casing and door, and serves to store the sediment sample (Fig. 8a). The corer has two tapered windows measuring 14 cm in length on each of the four sides to allow the researcher to examine how much lake sediment is collected (Fig. 8a).

**Fig. 10:**
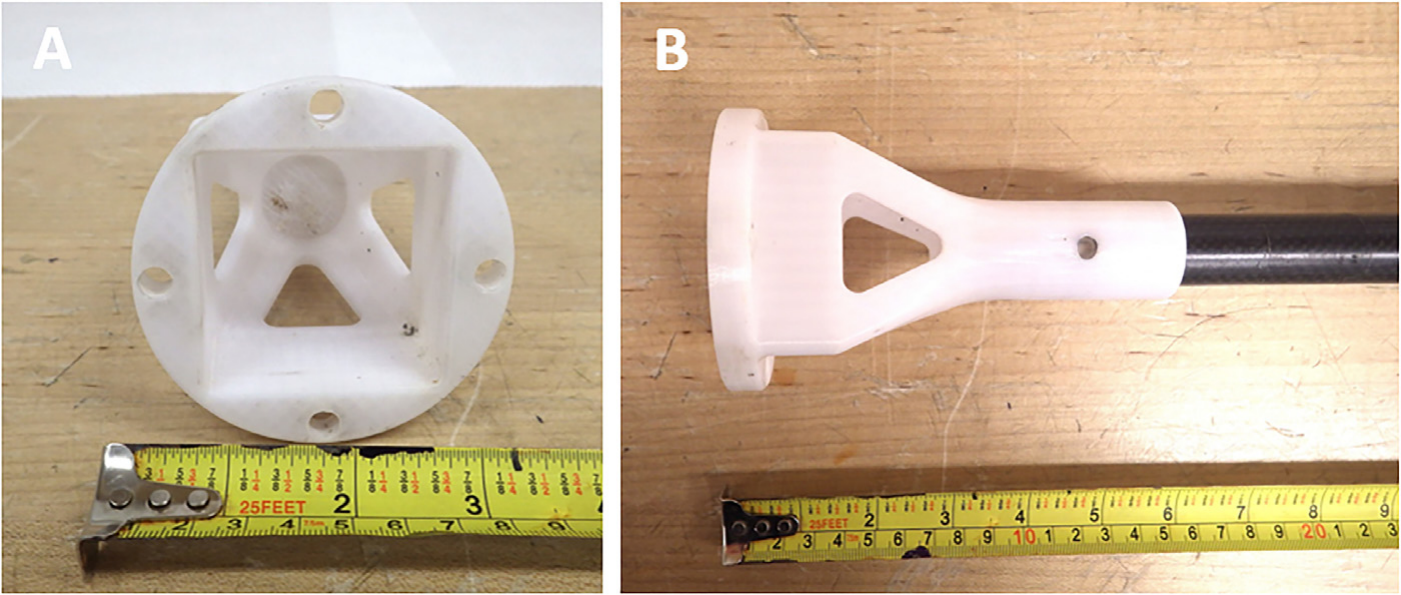
Lake sediment corer sampling attachment (a) secured to carbon fiber tubing (b).

The opposite end includes a circular platform (8.0 × 8.0 × 1.0 cm) where cylindrical openings (1.7 × 1.7 × 1.0 cm) for four stainless steel threads are installed (Fig. 8b). A 5.4 × 5.4 cm square opening is present allowing the acrylic tubing to be loaded into the corer (Fig. 8b). The circular platform is attached to the sampling pole (Fig. 10) using stainless steel screws. The sampling pole consists of a plastic attachment measuring 12 × 8.0 × 0. 4 cm (Fig. 10). The plastic attachment is structured similarly to the sediment corer in Fig. 8b, except that there is 3 cm of space for the acrylic tubing to be secured inside the plastic attachment once connected to the sediment corer. The plastic attachment is tapered in four compass directions at an angle of 45° for 3 cm and forms a cylindrical shape measuring 8.0 × 0.4 × 2.9 cm (Fig. 10b). A hole 0.6 cm wide is drilled perpendicular to the cylinder and carbon fibre tubing (2 cm diameter), and the carbon fibre tubing is inserted inside the plastic cylinder (Fig. 10b). A stainless steel screw is threaded through both the plastic attachment and carbon fiber tubing, connecting each portion (Fig. 10b). The sampler length can be extended by creating segments of carbon fibre tubing as described in section 2.8. Holes are drilled at each end and the segments can be attached using stainless steel cotter pins. The depth that the sediment corer can reach ranges from 1 to 5 m, enabling samples to be taken from watercraft or over ice in ponds and shallow lakes.

**Fig. 11:**
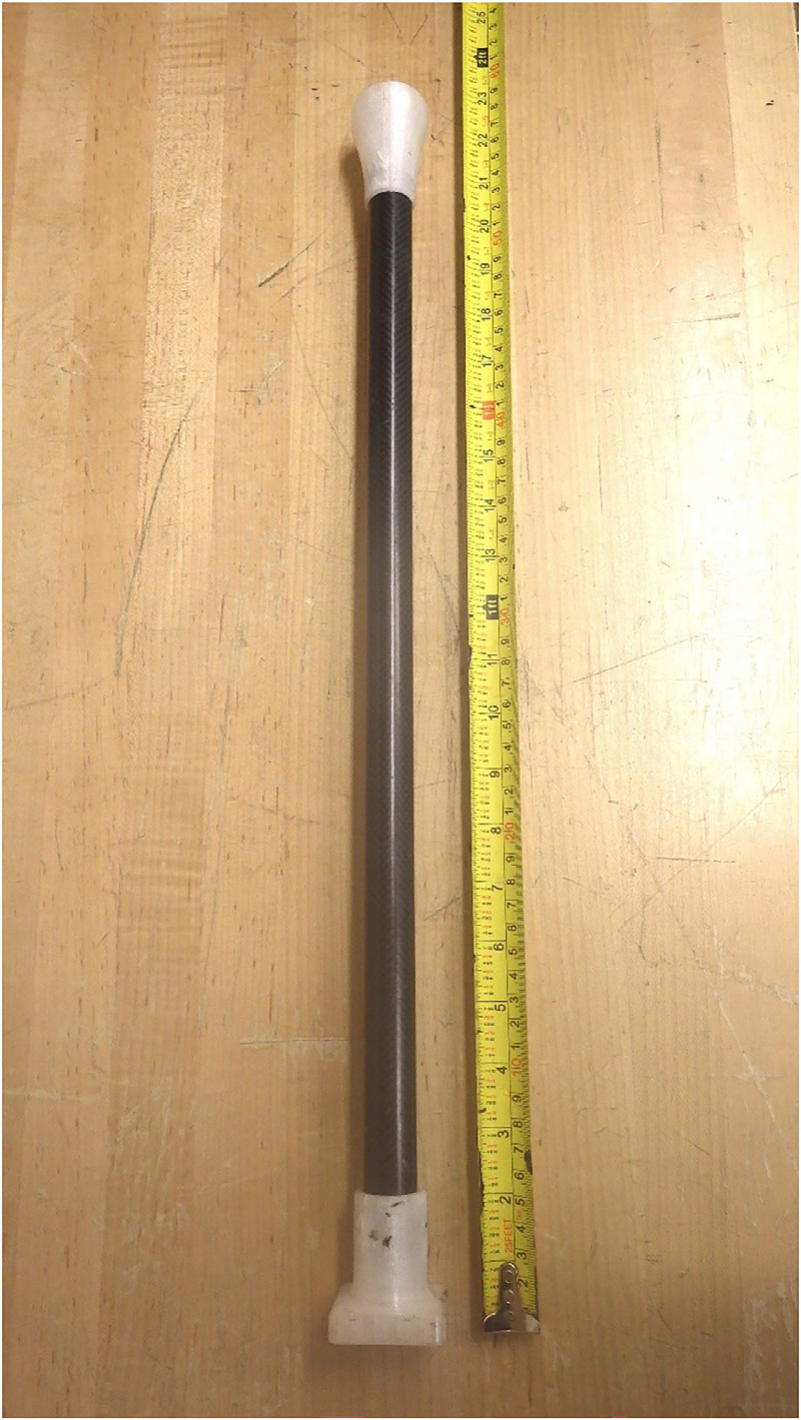
Square tool used for removing acrylic tubing housing a sediment sample from the lake sediment corer.

Furthermore, a plastic tool 58 cm in length was developed to remove the sediment sample in the acrylic tubing from the corer into separate bottles (Figs. 10; 11). The tool can also be used to push the acrylic tubing out of the corer (Figs. 10; 11). The plastic tool features a square plastic end piece with the square portion measuring 1.0 × 4.4 × 4.4 cm (Fig. 11). The four corners of the square end piece tapers down to form a cylinder (3.0 × 0.4 × 4 cm) that is pushed onto carbon fibre tubing (Fig. 11). On the opposite end of the tool is a circular plastic end piece (5.8 cm in length), with the larger circular portion measuring 1.4 × 3.2 × 3.2 cm (Fig. 4). This end serves as the handle, and tapers into a narrower cylinder (Fig. 11). To remove the sediment from the sampler, the sediment corer must first be disassembled from the carbon fiber pole, leaving the circular end of the corer open (Figs. 8; 10). The square end piece is inserted into the tapered 4.2 × 4.2 cm square end of the sediment corer and the plastic tool is used to push the sediment out towards the circular end of the corer (Figs. 8a; 11). Using clean sampling and handling methods [42], the sediment sample is pushed from the corer and emptied into plastic bottles. Alternatively, the sediment can be stored in the acrylic tubing. Two square PP pieces (4.5 × 4.5 × 0.5 cm) can be inserted inside the acrylic tubing to prevent the sample from shifting during transport and is wrapped with plastic wrap (Fig. 9d).

### Validation of the metal-free characteristic of the devices

All of the plastic materials used in our laboratory and described herein are subjected to simple leaching tests to identify any anomalous concentrations of TEs. Specifically, a 5 cm strand of each plastic weighing approximately 150 mg is cleaned using our routine SWAMP laboratory cleaning procedure for plasticware [46], then leached for 24 h in 10 ml of 2% nitric acid; the acid is purified twice by sub-boiling distillation in a quartz still (Duopur, MLS, Leutkirch, Germany). Leachates are analyzed for approximately 40 TEs using ICP-MS (iCAP Qc, ThermoFisher Scientific, Waltham, MA, USA). Cleaning, leaching, and analysis all takes place in the metal-free, ultraclean, SWAMP laboratory. These leaching conditions are extremely corrosive (pH < 1), in contrast to the pH range of most natural waters (pH 4 to 8). We do not follow any of the diverse, commonly used, standardized methods for quantifying element migration, extraction, or leaching (see [55], for a review). However, the purpose of our tests is simply to identify whether there are anomalous concentrations of individual TEs in the plastics that might contribute significantly to blank concentrations. Examples of such anomalous element releases include leaching of Pb from LDPE, Sb from PET, V from HDPE, or Zn from rubber (Table S2).

## Disadvantages of metal-free sampling equipment

Using metal-free sampling equipment that has been cleaned in high purity acid allows the greatest risks of TE contamination to be effectively eliminated or at least dramatically reduced [2,^4^,^11^,^15^,^17^,[19], [20], [21],[23], [24], [25]. All of the equipment described here has been shown to function properly for its intended application. However, some disadvantages exist and need to be acknowledged. Firstly, the majority of the metal-free sampling equipment described here were fabricated using PETG and PP. Unlike the metal alloys used in many sampling devices, these 3-D printed materials are not durable and are prone to breakage if not handled with care. Cold weather amplifies this problem, as it was observed that certain plastic sampling devices became more brittle when exposed to freezing temperatures. For example, the sediment corer was used during winter sampling, and freezing would occur around the 316 stainless steel threads (Fig. 8b). After repeated sampling, water freezing around the threads caused the plastic to be more brittle and would crack, which presented a problem in ensuring the sampler was properly secured to the pole. Secondly, any of the equipment mounted on aluminum poles (e.g. the rain and dust collectors) must be securely fixed to the ground with ropes and tent pegs (Figs. 1; 3). High winds can cause the rain and dust collectors to dislodge from the pole and result in damage if they are not secured properly. Thirdly, carbon fibre tubing used for the sediment corer and plant collectors is lightweight, but not indestructible; during sediment coring, cracking around the holes connecting the carbon fibre tubing segments was observed when sampled from a watercraft. It is advisable to purchase high quality carbon fibre tubing for this device, and that the sediment corer and plant collector are handled with care. And lastly, the sediment corer is ideal for fine-grained sediments, but certainly not for gravel or cobbles. Small rocks and pebbles create difficulty in attaining a sediment core as these obstacles prevent the corer from easily descending into the sediment. The corer must be handled with care in these conditions, or the plastic will crack.

## Summary

Metal-free devices help to mitigate TE contamination risks when sampling dust, rainwater, surface waters, plants, and sediments. Our laboratory has been able to construct metal-free devices that are not available through commercial means while providing invaluable innovations for environmental TE research. Metal-free devices allows researchers to mitigate a major source of contamination: sample collection. The use of metal-free equipment helps to reduce TE blank values, thereby improving analytical sensitivity, accuracy and precision. This is crucial for understanding the sources, transformation, and speciation of various TEs, and to assess their relevance for environmental and human health.

## Ethics statement

This material is the authors’ own original work and is truthful and complete. The results are appropriately placed in the context of prior and existing research. All authors have been personally and actively involved in substantial work leading to the paper and will take responsibility for its content. The paper properly credits the meaningful contributions of co-authors and co-researchers. All sources used are properly disclosed using correct citation.

## Funding

This work was supported by Alberta Innovates, Canada's Oil Sands Innovation Alliance (COSIA), the Natural Sciences and Engineering Research Council (NSERC), Suncor Energy Inc., and the SWAMP laboratory.

## CRediT authorship contribution statement

**Tommy Noernberg:** Conceptualization, Project administration, Writing – review & editing, Validation. **Taylor Bujaczek:** Validation, Writing – original draft. **Chad W. Cuss:** Investigation, Project administration, Validation. **William Shotyk:** Conceptualization, Methodology, Supervision, Writing – original draft, Writing – review & editing, Project administration, Funding acquisition.

## Data Availability

Data will be made available on request.

## Appendix Supplementary materials

Image, application 1
