# Supplementary material for: Metal-free sampling methods for dust, rainwater, surface water, plants, and sediments: A selection of unique tools from the SWAMP laboratory
Source: MethodsX. 2023 Dec 20;12:102521. doi: 10.1016/j.mex.2023.102521 (PMC10787232; doi:10.1016/j.mex.2023.102521)
Supplement: Supplementary file 1 [file mmc1.docx]

***Supplementary Information for***

Metal-free sampling methods for dust, rainwater, surface water, plants, and sediments:

a selection of unique tools from the SWAMP laboratory

Tommy Noernberg^1^, Taylor Bujaczek^1^, Chad W. Cuss^1,2^, and William Shotyk^3†^

^1^ Department of Renewable Resources, University of Alberta, Edmonton, AB, Canada T6G 2R3

^2^ Current affiliation: School of Science and the Environment, Grenfell Campus, Memorial University of Newfoundland, Corner Brook, NL, Canada A2H 5G4

^3^ Bocock Chair for Agriculture and the Environment, Department of Renewable Resources, University of Alberta, Edmonton, AB, Canada T6G 2H1

† Corresponding author: William Shotyk ([shotyk@ualberta.ca](file:///C:\Users\shotyk\AppData\Local\Microsoft\Windows\Temporary%20Internet%20Files\Content.Outlook\W7N38TUY\shotyk@ualberta.ca))

Department of Renewable Resources, University of Alberta, 348B South Academic Building, Edmonton, Alberta CANADA T6G 2H. Tel.: 780-492-7155 Fax: 780-492-4323

REVISED MANUSCRIPT For submission to **Methods-X**

**Contents**

**List of Tables**

**Table S1:** Metal-free devices for environmental sampling designed and constructed at the SWAMP laboratory including their intended use, plastic type, and any metal alloys used.

**Table S2:** Materials to avoid in sampling devices because of potential leaching of specific TEs.

**Table S3:** Particle count performed outside and inside of the three HEPA filtered growth chambers housed within the greenhouse facility in the Faculty of Life, Agricultural and Environmental Sciences at the University of Alberta.

**Table S1:** Metal-free devices for environmental sampling designed and constructed at the SWAMP laboratory including their intended use, plastic type, and any metal alloys used.

| **Device** | **Intended Sample Media** | **Plastic** | **Metal Alloy** |
| --- | --- | --- | --- |
| Aeolian Frisbee dust collector | Dust | PETG, PP | Aluminum 6061 |
| Moss bag | Dust | PETG | --- |
| Moisture activated rain collector | Rainwater | PETG | 316 stainless steel |
| Water column sampler | Surface Water | PETG | 316 stainless steel |
| SWAMP Lab “Fish” | River and Stream Water | PETG | 316 stainless steel |
| Laminar flow, HEPA-filtered growth chambers | Plant | Vinyl | Anodized aluminum 6560 |
| Aquatic plant collector | Plant | PETG | 316 stainless steel, aluminum 6061 |
| Sediment corer | Sediment | PETG | 316 stainless steel, aluminum 6061 |

**Table S2:** Materials to avoid in sampling devices because of potential leaching of specific TEs.

| **Material** | **TE Leached** | **Reference** |
| --- | --- | --- |
| Brass | Ag, As, Cu, Sb, Tl, Zn | (1) |
| Steel | Cd, Pb, Zn | (1) |
| Titanium alloys | Pb | (1) |
| Pewter | Sb, Sn | (2) |
| Glass | As, Pb, Sb, Th, Zn | (2), (3), (4) |
| Rubber | Zn | (1) |
| Polyethylene terephthalate | Sb | (5), (6) |
| High density polyethylene | Al, V | (1, 7) |
| Low density polyethylene | Pb | (1) |
| Mixed plastics (e.g. recycled) | Sb, V | (8) |
| Polyvinyl chloride | Cd, Pb | (9), (10) |

**Table S3:** Particle count performed outside and inside of the three HEPA filtered growth chambers housed within the greenhouse facility in the Faculty of Life, Agricultural and Environmental Sciences at the University of Alberta.

| Particle size (µm) | Number of particles/ft^3^ | | | |
| --- | --- | --- | --- | --- |
|  | Outside the chambers | Inside 1 | Inside 2 | Inside 3 |
| 0.3 | 124,890 | 0 | 0 | 0 |
| 0.5 | 12,170 | 0 | 0 | 0 |
| 1.0 | 1,400 | 0 | 0 | 0 |
| 2.5 | 330 | 0 | 0 | 0 |
| 5.0 | 0 | 0 | 0 | 0 |
| 10.0 | 10 | 0 | 0 | 0 |

**References**

1. W. Shotyk, B. Bicalho, C.W. Cuss, I. Grant-Weaver, M.B. Javed, M. Krachler, T. Noernberg, M.A. Powell, J. Zheng, (in-press) The Elmvale Groundwater Observatory: A Facility Developed to Sample Pristine, Artesian Groundwaters for Trace Elements, 1^st^ ed. The Groundwater Project, Guelph, 2023.
2. W. Shotyk, M. Krachler, Trace and ultratrace metals in bottled waters: survey of sources worldwide and comparison with refillable metal bottles, *Sci. Total Environ.* 407(3) (2009) 1089-1096, doi: 10.1016/j.scitotenv.2008.10.014.
3. C. Furneisen, Arsenic in bottled waters, Diploma thesis, University of Heidelberg, 2010.
4. W. Shotyk, M. Krachler, Lead in bottled waters: comparison with pristine groundwaters and contamination from glass, *Environ. Sci. Technol.* 41(10) (2007) 3508-3515, doi: 10.1021/es062964h.
5. W. Shotyk, M. Krachler, B. Chen, Contamination of Canadian and European bottled waters with antimony leaching from PET containers, *J. Environ. Monitor.* 8(2) (2006) 288-292, doi: [10.1039/b517844b](http://dx.doi.org/10.1039/b517844b).
6. W. Shotyk, M. Krachler, Contamination of bottled waters with antimony leaching from PET increases with storage, *Environ. Sci. Technol.* 41(5) (2007) 1560-1563, doi: 10.1021/es061511+.
7. W. Shotyk, B. Bicalho, C.W. Cuss, M. Donner, I. Grant-Weaver, S. Haas-Neill, M. Javed, M. Krachler, T. Noernberg, R. Pelletier, Z. Zaccone, Trace metals in the dissolved fraction (< 0.45 µm) of the lower Athabasca River: analytical challenges and environmental implications. *Sci. Total Environ.* 580 (2017) 660-669, doi: 10.1016/j.scitotenv.2016.12.012.
8. B. Chen, M. Krachler, W. Shotyk, Determination of antimony in plant and peat samples by hydride generation – atomic fluorescence spectrometry (HG-AFS), *J. Anal. Atom. Spectrom*. 18(10) (2003) 1256-1262, doi: 10.1039/b306597a.
9. M.A. Belarra, J.M. Anzano, J.R. Castillo, Determination of lead and cadmium in samples of poly(vinyl chloride) using organic solvents by flame atomic absorption spectrometry. *Z. Anal. Chem.* 334(2) (1989), 118-121, doi: 10.1007CF00476669.
10. M.A. Belarra, J.M. Anzano, J.R. Castillo, Determination of lead and cadmium in samples of poly(vinyl chloride) by electrothermal atomic absorption spectrometry using organic solvents, *Analyst*, 115(7) (1990) 955-957, doi: 10.1039/AN9901500955.
